# Resveratrol-Loaded Albumin Nanoparticles with Prolonged Blood Circulation and Improved Biocompatibility for Highly Effective Targeted Pancreatic Tumor Therapy

**DOI:** 10.1186/s11671-017-2206-6

**Published:** 2017-06-30

**Authors:** Tao Geng, Xia Zhao, Meng Ma, Gang Zhu, Ling Yin

**Affiliations:** 1 Department of Pharmacy, the Affiliated Hospital of Taishan Medical University, Tai’an, 271000 China; 2 Department of Pharmacy, Shandong Qianfoshan Hospital, Jinan, 250000 China; 3 Tai’an Maternal and Child Health Hospital, Tai’an, 271000 China; 4 Taishan People’s Hospital, Tai’an, 271000 China; 5 Affiliated Hospital of Taishan Medical University, Tai’an, 271000 China

**Keywords:** Human serum albumin, Resveratrol, Blood circulation, Biocompatibility, Anti-cancer

## Abstract

Human serum albumin (HSA) is an intrinsic protein and important carrier that transports endogenous as well as exogenous substances across cell membranes. Herein, we have designed and prepared resveratrol (RV)-loaded HSA nanoparticles conjugating RGD (arginine–glycine–aspartate) via a polyethylene glycol (PEG) “bridge” (HRP–RGD NPs) for highly effective targeted pancreatic tumor therapy. HRP–RGD NPs possess an average size of 120 ± 2.6 nm with a narrow distribution, a homodisperse spherical shape, a RV encapsulation efficiency of 62.5 ± 4.21%, and a maximum RV release ratio of 58.4.2 ± 2.8% at pH 5.0 and 37 °C. In vitro biocompatibility of RV is improved after coating with HSA and PEG. Confocal fluorescence images show that HRP–RGD NPs have the highest cellular uptake ratio of 47.3 ± 4.6% compared to HRP NPs and HRP–RGD NPs with free RGD blocking, attributing to an RGD-mediated effect. A cell counting kit-8 (CCK-8) assay indicates that HRP–RGD NPs without RV (HP–RGD NPs) have nearly no cytotoxicity, but HRP–RGD NPs are significantly more cytotoxic to PANC-1 cells compared to free RV and HRP NPs in a concentration dependent manner, showing apoptotic morphology. Furthermore, with a formulated PEG and HSA coating, HRP–RGD NPs prolong the blood circulation of RV, increasing approximately 5.43-fold (t_1/2_). After intravenous injection into tumor-bearing mice, the content of HRP–RGD NPs in tumor tissue was proven to be approximately 3.01- and 8.1-fold higher than that of HRP NPs and free RV, respectively. Based on these results, HRP–RGD NPs were used in an in vivo anti-cancer study and demonstrated the best tumor growth suppression effect of all tested drugs with no relapse, high in vivo biocompatibility, and no significant systemic toxicity over 35 days treatment. These results demonstrate that HRP–RGD NPs with prolonged blood circulation and improved biocompatibility have high anti-cancer effects with promising future applications in cancer therapy.

## Background

Pancreatic cancer represents a devastating disease with less than 6 months of median survival and only a 5-year survival rate of 6% [1]. The traditional clinical treatment for pancreatic cancer is surgical excision, radiotherapy, and chemotherapy [2, 3]. However, these methods may be limited by serious side effects, such as the spreading of cancer cells after incomplete excision, serious toxicity to normal cells during radiotherapy, and poor survival rates [4]. Although low target effects and high side effects have also limited the utility of anti-cancer drugs in chemotherapy, increasingly new chemotherapeutic agents are being developed. Apart from synthetic drugs, many Chinese herb extracts are found to be effective against certain cancer types. Resveratrol (RV), a natural extractive from vegetation such as grapes and soy beans [5], has been widely acted in platelet aggregation and inhibiting vasodilation, and reducing blood viscosity [6, 7]. And in recent decades, it has also been found to have great anti-cancer effects in some cancers, such as liver, breast, and ovarian cancer [8, 9]. However, utilizing RV as a potential anti-cancer drug has some drawbacks for further clinical application, such as poor solubility, low blood circulation, and lack of selectivity [4, 10].

Under the premise of protecting the structural integrity of the drug, encapsulation strategy has attracted many researchers’ interest, which has been demonstrated to be effective in overcoming some of the abovementioned drawbacks compared to conventional “free” drugs [11]. For instance, it can improve poor solubility and low bioavailability, lower fast renal clearance, as well as increase cells selectivity [12]. Currently, many encapsulation methods such as by liposomes, polymeric-based nanoparticles, hydrogels, and serum albumin are used [13–16]. Among these methods, the use of serum albumin has become one of the most exciting carriers to deliver insoluble anti-cancer drugs. Human serum albumin (HSA), an endogenous protein is non-toxic, shows non-immunogenicity and has great biocompatibility [17]. It has been widely used as a macromolecular protein carrier for drug delivery [18]. Thus, HSA is able to improve the solubility of lipophilic drugs. Moreover, the presence of functional carboxylic and amino groups on the surface facilitates the surface functionalization for albumin nanoparticles [19, 20]. For example, via covalent binding, the surface of albumin nanoparticles can be decorated with fluorescence dyes, target molecules, and functional RNA [21, 22]. Also, it can be readily functionalized with hydrophilic polymers, such as PEG, to prolong the blood circulation [23].

In this study, HSA is used to encapsulate lipophilic RV as a nanodrug which is surface functionalized with a tumor targeting molecule, arginine–glycine–aspartate (RGD) via a PEG “bridge” (HRP–RGD NPs). The prepared HRP–RGD NPs demonstrate great in vitro and in vivo biocompatibility, and prolonged blood circulation. The cell uptake and in vivo tumor biodistribution were also evaluated to validate its targeting potential in PANC-1 cells. Moreover, the targeted anti-cancer efficacy of HRP–RGD NPs was investigated in vitro and in vivo. These results indicate that HRP–RGD NPs may be a versatile nanoplatform for potential tumor therapeutic agents for targeted chemotherapy application.

## Methods

### Materials

Human serum albumin (HSA, lyophilized powder, ≥96%), resveratrol (RV, ≥99%), 1-ethyl-3-(3-dimethylaminopropyl) carbodiimide (EDC), fluorescein isothiocyanate (FITC), Arg–Gly–Asp (RGD, ≥97%), 3-(2-pyridyldithio) propionic acid *N*-hydroxysuccinimide ester (SPDP), Hoechst 33258 dye, and 4′,6-diamidino-2-phenylindole (DAPI) were obtained from Sigma Aldrich (St. Louis, MO, USA). NH_2_–PEG_2000_–COOH was bought from Seebio Biotech Inc. (Shanghai, China). *N*-Succinimidyl *S*-acetylthioacetate (SATA) was purchased from Pierce Biotech Inc. (Rockford, IL, USA). DMSO, Trypsin–EDTA solution, phosphate buffer (PBS), fetal bovine serum (FBS), penicillin–streptomycin solution, and DMEM media were purchased from Sigma.

### Synthesis of HSA–RV Nanoparticles

HSA–RV nanoparticles were synthesized by a simple desolvation method [24]. In detail, 6 mg RV was dissolved in DMSO to be 1 mg/mL and was mixed with 10 mg of HSA in 1 mL water under slighted stirring, forming hardened coacervates after stirring for 6 h under room temperature, and then was processed by cross-linking with 0.5% glutaraldehyde (100 μL). Afterwards, the organic solvents were removed by dialyzing in water for 1 day, resulting in the HSA–RV nanoparticles. Blank HSA nanoparticles were prepared as mentioned above, except that DMSO without RV was mixed with HSA solution for 6 h.

### Synthesis and Characterization of HRP–RGD NPs

HSA–RV nanoparticles were conjugated with HS–PEG–RGD by the traditional cross-linker SPDP as described in literatures [25]. In brief, 20 mg NH_2_–PEG_2000_–COOH was treated with 2 mg SATA for 3 h and purified by desalting. The resulted SATA–PEG was added into a 10 mg RGD peptide and 8 mg EDC for 3 h. The SATA-protected PEG–RGD was then reacted with 1 mL hydroxylamine in sodium phosphate for 3 h. After purified by desalting, it was given the HS–PEG–RGD. Next, the obtained HS–PEG–RGD was conjugated with HSA–RV nanoparticles via disulfide linkages. Briefly, the amino groups of the HSA–RV nanoparticles were firstly activated by SPDP and then were re-suspended in 10 mL PBS and reacted with excess HS–PEG–RGD or HS–PEG for 24 h. The resulted solution was repeated washed by PBS and filtered through a Millipore filter (100 kDa) to remove any remaining PEG–RGD and other organic solvents, resulting in HRP–RGD NPs. The morphology and size of the nanoparticles were detected by scanning electron microscopy (SEM, Philips XL-30 FEG, Eindhoven, Netherlands) and a Zetasizer Nano ZS system (Malvern Instruments, Malvern, UK), respectively. Absorption spectra were acquired by a UV–Vis spectrophotometer (UV1800, Shimadzu, Japan). Fluorescence spectra were recorded by a fluorescence spectrometer (F-4500, Hitachi, Japan).

### Drug Loading and Release

Six milligrams of RV was dissolved in DMSO to be 1 mg/mL and was mixed with 10 mg of HSA in 1 mL water under slighted stirring, forming hardened coacervates after stirring for 6 h under room temperature, and then was processed by cross-linking with 0.5% glutaraldehyde (100 μL). Afterwards, the organic solvents and free RV were removed by dialyzing in water for 1 day. The dialyzate was used to quantify the free RV by UV–Vis spectrometer at 306 nm according to a calibration curve. The amount of drug in HRP–RGD NPs was total added RV minus the amount of free RV.

RV release from HRP–RGD NPs was detected via a dynamic dialysis technique (dialysis bag with a cutoff Mw of 8–12 kDa) at pH 5.0, 7.4 and 9.0 PBS, respectively, at 37 °C. The drug concentration was calculated using a standard calibration curve. The encapsulation efficiency = *W*_1_/*W*_2_ × 100%, where *W*_1_ represents the weight of RV in HRP–RGD NPs, and *W*_2_ is the weight of RV added. Cumulative release = *W*_a_/*W*_b_ × 100%, where *W*_a_ represents the amount of RV released, and *W*_b_ is the total RV present in HRP–RGD NPs.

### In Vitro Hemolysis Assay

In vitro hemolysis assay was conducted as described in previous work [26]. In detail, 0.2 mL red blood cells (RBCs, in PBS) were mixed with 0.8 mL HRP–RGD NPs (in PBS) at predetermined concentrations (10, 50, 100, and 200 μg/mL). RBCs incubated with deionized water or PBS were set as positive or negative control, respectively. After incubated at 37 °C for 3 h, the above set of suspensions were centrifuged at 10,000 rpm for 1 min and the absorbance of the supernatants at 541 nm was monitored by a UV–Vis spectrometer. Hemolytic ratio = (OD_t_ − OD_nc_)/(OD_pc_ − OD_nc_) × 100%, where OD_t_, OD_pc_, and OD_nc_ are the absorbance of the supernatant of the test sample, positive and negative controls, respectively.

### Cell Culture

Human pancreatic tumor PANC-1 cells were purchased from the Cell Bank of Type Culture Collection of Chinese Academy of Sciences (Shanghai, China) and cultured in DMEM media supplemented with 10% FBS and 1% penicillin–streptomycin at a humidified incubator (5% CO_2_) at 37 °C.

### Cellular Uptake

For cellular uptake, FITC was used to label the HRP–RGD NPs. PANC-1 cells adhered to glass slides in 6-well plates and were incubated with HRP NPs, HRP–RGD NPs + free RGD blocking, and HRP–RGD NPs at the same concentration of labeled FITC for 5 h, respectively. And then, the cells were washed with PBS thrice and fixed by 0.2 mL of glutaraldehyde, followed by staining with DAPI for 10 min. The fluorescence images of cells were captured by the laser scanning confocal microscope (Leica TCS SP8 CARS, Wetzlar, Germany).

In addition, the uptake ratios of HRP NPs, HRP–RGD NPs + free RGD blocking, and HRP–RGD NPs at the same concentration of labeled FITC by PNAC-1 cells were analyzed by using flow cytometry (FCM, FACSCalibur, FACSCanto II) through measuring FITC fluorescence. Ten thousands cells were recorded for each FCM analysis. The FITC fluorescence was excited using a 488-nm laser.

### In Vitro Cytotoxicity

The cytotoxicity of HP–RGD NPs, the carrier of RV, was conducted by using a standard cell counting kit-8 (CCK-8) assay (Bestbio, China). PNAC-1 cells (1 × 10^5^ cells/mL, 0.5 mL) were seeded in 96-well plate and cultured for 24 h. After discarding the old media, fresh media containing 10, 50, 100 and 200 μg/mL of HRP–RGD NPs were incubated with PNAC-1 cells for 24 h. PBS was used to mildly wash the cells three times. A 100 μL CCK-8 working solution (10% CCK-8 + 90% DMEM) was then added to each well, followed by incubation at 37 °C for 0.5 h. The absorbance value at 450 nm was detected using a microplate reader (Infinite 200 Pro, Tecan, Austria). Furthermore, the in vitro anti-cancer efficacy of the RV (dissolved in DMSO), HRP NPs, and HRP–RGD NPs with the same RV concentration against PNAC-1 cells was evaluated by CCK-8 assay mentioned above. All experiments were performed in quadruple occasions. In addition, the morphological examination for apoptosis was detected by Hoechst 33258 staining. The fluorescence of Hoechst 33258 in cells were observed and recorded by laser scanning confocal microscope.

### Animal Model

Balb/c nude mice, 4–5 weeks, were purchased from the Shanghai Slac Laboratory Animal Co. Ltd. (Shanghai, China). All animal experiments were performed in accordance with the Guide for the Care and Use of Laboratory Animals and approved by the Taishan Medical University Administration Office of Laboratory Animal. Subcutaneous tumor xenograft models were established in the right back region of mice by injecting 1 × 10^6^ PNAC-1 cells per mouse, and when the tumors exhibited a volume of about 80 mm^3^, these mice were randomly divided into different groups (*n* = 5) for further use.

### Blood Circulation and Tumor Biodistribution

The normal mice were intravenous injected with RV, HRP NPs, and HRP–RGD NPs. Afterwards, blood samples were collected at different time from the orbital plexus. Each blood sample was dissolved in 900 μL of lysis buffer. The concentration of RV, HRP NPs, and HRP–RGD NPs in the blood was determined by RV absorbance spectra of each solubilized blood sample by an UV–Vis spectrometer. The sample concentrations are defined as the percentage of injected dose per gram of tissue (ID%/g).

Biodistribution in tumor was performed in tumor-bearing mice. The tumor tissues were weighed and digested by aqua regia solution overnight at 24 h post intravenous injection of RV, HRP NPs, and HRP–RGD NPs, respectively. The concentration of RV, HRP NPs, and HRP–RGD NPs in the tumor was determined by RV absorbance spectra of each solubilized tumor tissue by an UV–Vis spectrometer. The sample concentrations are defined as the percentage of injected dose per gram of tissue (ID%/g).

### In Vivo Anti-cancer Efficacy

The mice of different groups were injected intravenously with saline, RV, HRP NPs, and HRP–RGD NPs (the dose equal to RV, *n* = 5, 10 mg/kg). During the treatment, tumor sizes and body weights of the tumor-bearing mice were monitored every 3 days. The tumor volume was calculated using the formula: *V* = (length × width^2^)/2. Relative tumor volume = *V*/*V*_0_, where *V*_0_ was the tumor volume prior to the initial treatment.

### Histology Examination

Healthy Balb/c nude mice of different groups were intravenous injected with saline (control), RV, HRP NPs, and HRP–RGD NPs (the dose equal to RV, *n* = 5, 5 mg/kg). After 35 days, mice were sacrificed and the heart, liver, spleen, lung, and kidney were collected. The obtained major organs were fixed with 4% paraformaldehyde overnight. Afterwards, these organs were dehydrated in 25% sucrose, sectioned into 5 μm slices, and stained with hematoxylin and eosin (H&E). The stained sections were imaged under an inverted phase contrast microscope.

## Results and Discussion

### Synthesis and Characterization of HRP–RGD NPs

**Fig. 1:**
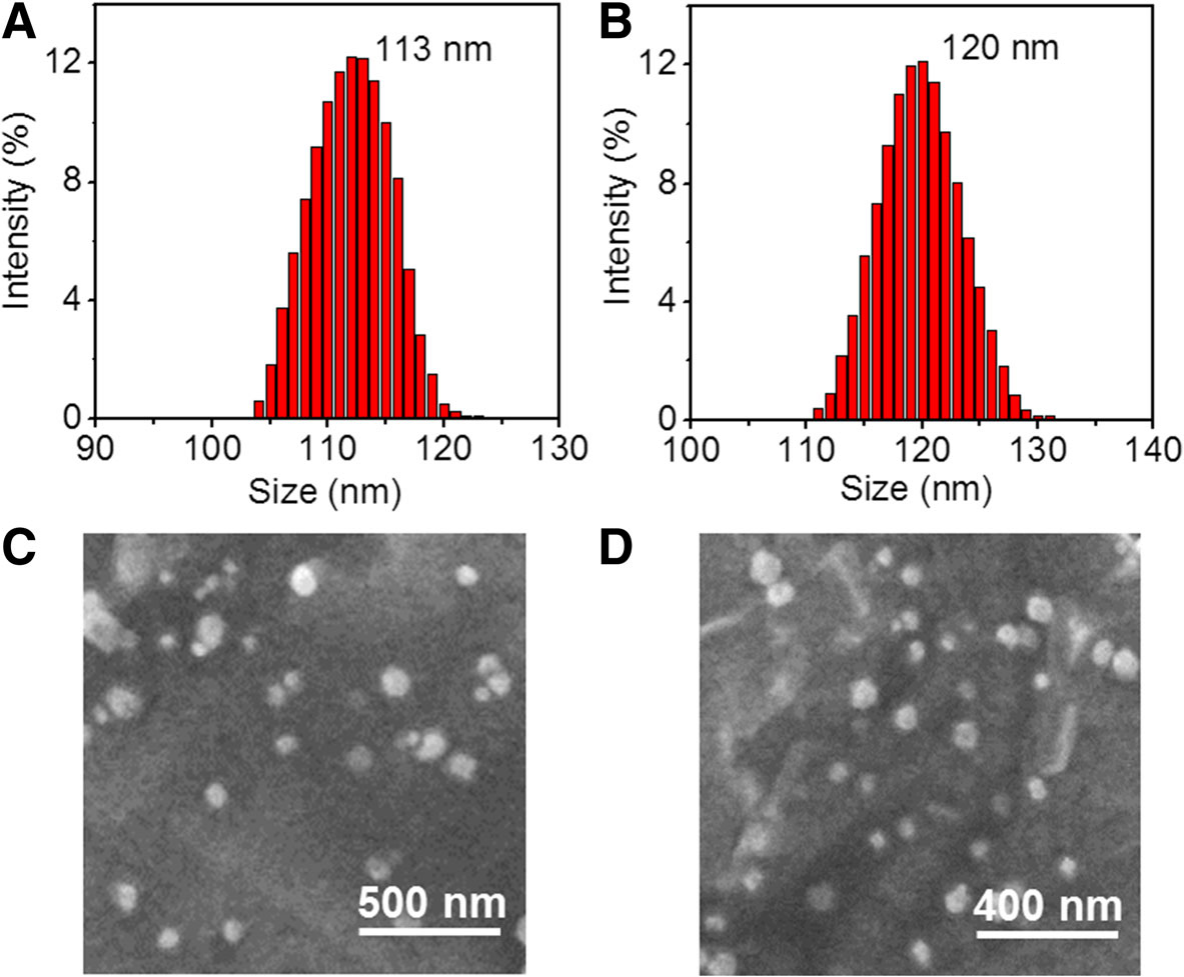
Particle size distribution of HRP NPs (**a**) and HRP–RGD NPs (**b**). SEM images of HRP NPs (**c**) and HRP–RGD NPs (**d**)

According to a simple desolvation method [24], RV was encapsulated by HSA, resulting in HSA–RV nanoparticles. After which, the surface of HSA–RV nanoparticles was covalently functionalized with HS–PEG–RGD by the traditional cross-linker SPDP [25], forming nanocomposite HRP–RGD NPs. The HRP NPs and HRP–RGD NPs showed unimodal and narrow particle size distribution (Fig. 1a, b). After conjugating RGD on the surface of HRP NPs, the average size of nanoparticles increased from 113 ± 3.1 nm to 120 ± 2.6 nm. Furthermore, HRP NPs and HRP–RGD NPs exhibited a homodisperse spherical-shaped morphology (Fig. 1c, d).

**Fig. 2:**
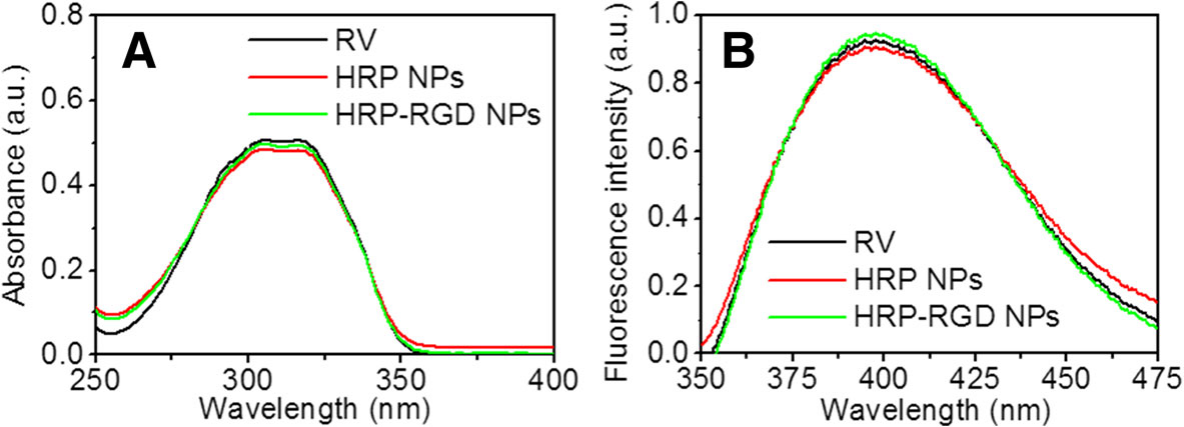
**a** The absorbance spectra of free RV, HRP NPs, and HRP–RGD NPs. **b** The fluorescence spectra of free RV, HRP NPs, and HRP–RGD NPs

UV–Vis and fluorescence spectra were taken to confirm the presence of RV in HRP–RGD NPs. As shown in Fig. 2a, HRP NPs and HRP–RGD NPs featured absorbance peaks for RV at 304 nm, indicating the presence of RV in both nanoparticles. In addition, HRP NPs and HRP–RGD NPs showed RV fluorescence signals at a 325-nm excitation wavelength, which is consistent with the fluorescence spectra of RV (Fig. 2b). These results demonstrate that RV retains its optical properties after conjugation to HRP NPs and HRP–RGD NPs.

### RV Loading and Release

**Fig. 3:**
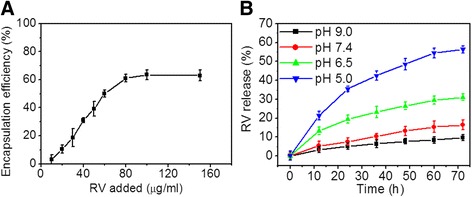
**a** RV encapsulation efficiency as the function of added RV concentrations. **b** In vitro release profile of RV from HRP–RGD NPs in pH 5.0, 6.5, 7.4, and 9.0 at 37 °C

Figure 3a shows the encapsulation efficiency change curve of RV in HRP–RGD NPs upon increasing RV concentration. The maximum RV EE of HRP–RGD NPs obtained was 62.5 ± 4.21%. In addition, as seen in Fig. 3b, HRP–RGD NPs exhibited the highest RV release rate of 58.4.2 ± 2.8% after 60 h at 37 °C and pH 5.0 compared to the release rate obtained at pH 6.5, pH 7.4, and pH 9.0 at 37 °C. It has been reported that the normal blood pH is 7.4, while tumor tissue is slightly acid [27]. This proves to be a beneficial feature of using HRP–RGD NPs in tumor therapy.

### In Vitro Biocompatibility

**Fig. 4:**
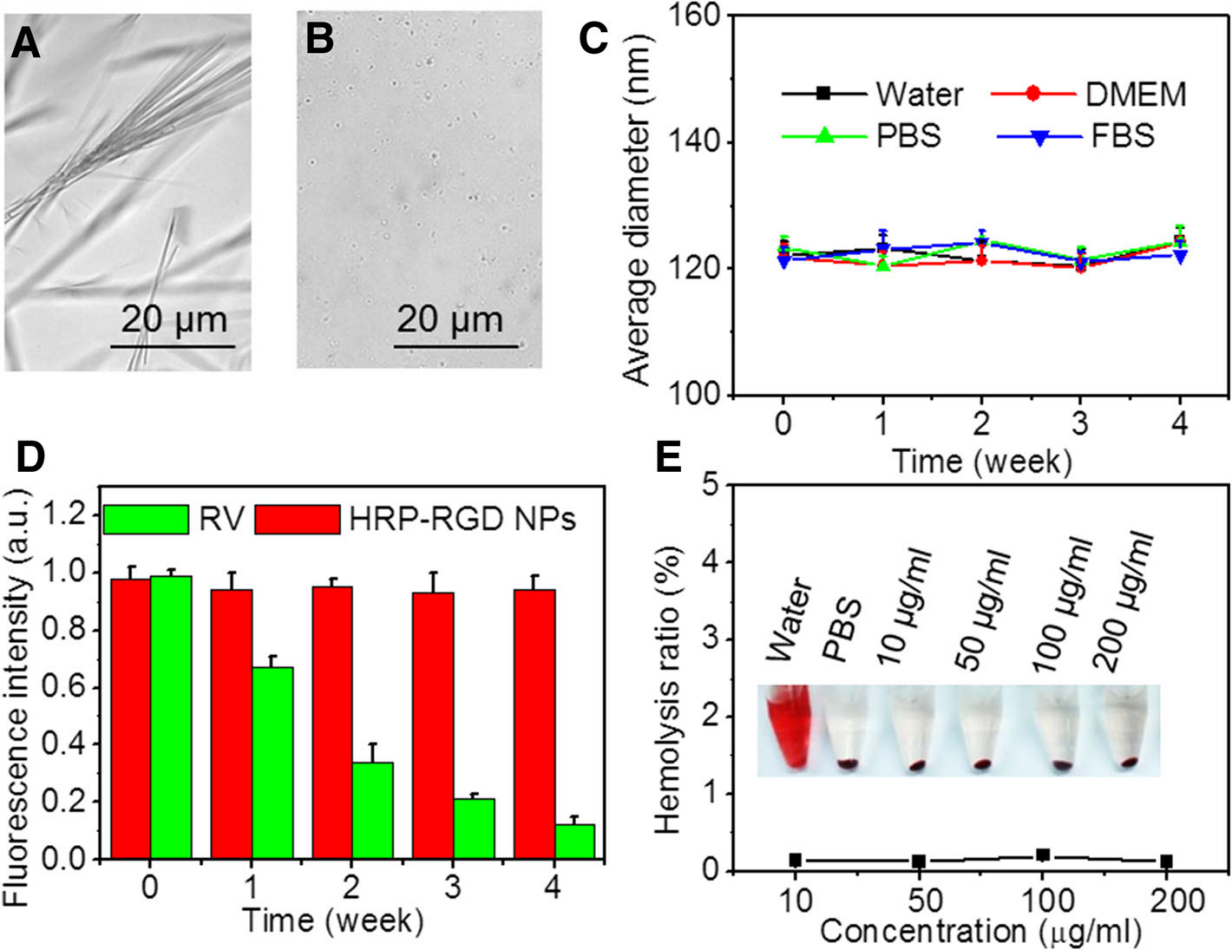
The microscope image of **a** free RV (dissolved in DMSO) in PBS and **b** HRP–RGD NPs in PBS after 7 days. **c** Colloid stability test of HRP–RGD NPs in different media (water, DMEM, PBS, and FBS). **d** The RV fluorescence stability in HRP–RGD NPs after 4 weeks. **e** Hemolysis ratio of RBCs after 1 h incubation with HRP–RGD NPs at different concentrations. The *inset* shows the photograph of RBCs exposed to distilled water, PBS, and HRP–RGD NPs with different concentrations followed by centrifugation

Figure 4a, b shows images of DMSO dissolved RV and HRP–RGD NPs in PBS at 4 °C after 7 days. The former shows solid matter like acicular crystal, while the latter has a lot of micro and homodisperse spherical particles, indicating the improved stability of RV after encapsulation by HSA nanoparticles. Furthermore, the average size of HRP–RGD NPs in water, DMEM, PBS, and FBS showed almost no variation over 4 weeks (Fig. 4c), demonstrating the high colloidal stability of HRP–RGD NPs, most likely ascribed to the PEG and HSA encapsulation.

Figures 4d shows the fluorescent stability of RV and HRP–RGD NPs in aqueous solution at 4 °C. After 4 weeks of storage, the RV fluorescence intensity of HRP–RGD NPs remained more than 96.8% of its initial intensities; however, the fluorescence of RV dropped rapidly to 12.1% of its initial intensity likely due to RV precipitation out of the solution [10], further indicating the stability of HRP–RGD NPs compared to free RV. Moreover, as shown in Fig. 4e, no significant hemolysis phenomenon was detected for HRP–RGD NPs-treated RBCs below 200 μg/mL, similar to that of the negative control PBS-treated group, illustrating the excellent hemocompatibility of HRP–RGD NPs. These results suggest that HSA encapsulation improved the stability and in vitro biocompatibility of RV, which is beneficial for biomedical applications.

### Cellular Uptake

**Fig. 5:**
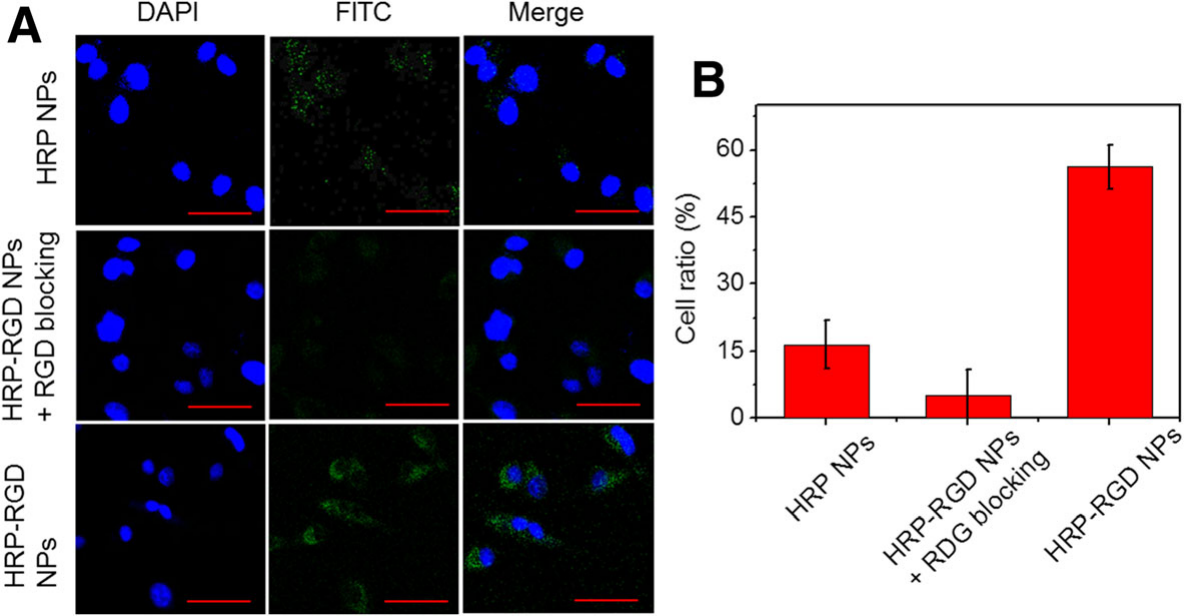
**a** Confocal fluorescence images of PANC-1 cells after incubation with HRP NPs, HRP–RGD NPs with RGD blocking, and HRP–RGD NPs labeled by FITC. *Green* and *blue colors* represent FITC fluorescence and DAPI stained cell nuclei, respectively. *Scale bar* = 20 μm. **b** The quantitative cellular uptake of PANC-1 cells towards HRP NPs, HRP–RGD NPs with RGD blocking, and HRP–RGD NPs by flow cytometry

HRP NPs and HRP–RGD NPs were labeled by FITC. As shown in Fig. 5a, the nuclei displayed blue fluorescence, which were stained by DAPI. An intense green fluorescence (FITC signal) was observed in the perinuclear region of PANC-1 cells treated with HRP–RGD NPs, showing that a sufficient amount of HRP–RGD NPs entered the cytoplasm. In contrast, very little green fluorescence was shown in HRP NPs-treated PANC-1 cells. Moreover, PANC-1 cells pre-treated with free RGD also exhibited mild green fluorescence, likely attributed to the RGD receptor on the PANC-1 surface being blocked by free RGD. The cellular uptake ratio of the nanoparticles was detected by FCM, which was 16.2 ± 4.9%, 7.1 ± 5.1%, and 58.5 ± 3.5% for HRP NPs, HRP–RGD NPs with RGD blocking, and HRP–RGD NPs-treated PANC-1 cells, respectively (Fig. 5b). These results demonstrate that the target molecule RGD can facilitate the high-efficiency uptake of HRP–RGD NPs by PANC-1 cells [28, 29].

### In Vitro Cytotoxicity

**Fig. 6:**
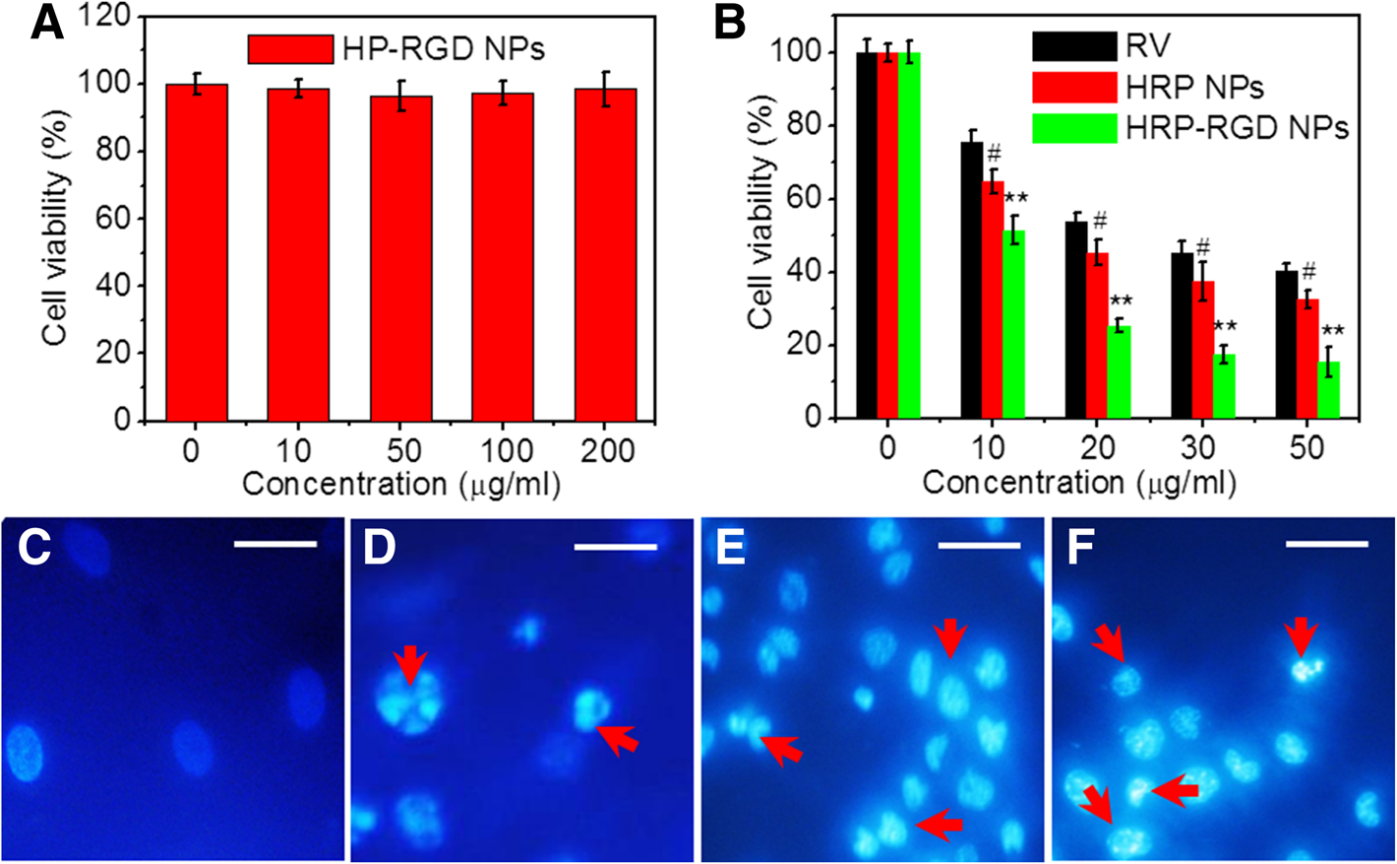
**a** Cell viability of PANC-1 cells after treatment with HP–RGD NPs for 24 h. **b** Cell viabilities of PANC-1 cells after incubation with different concentration of RV, HRP NPs, and HRP–RGD NPs. The nuclear of PANC-1 cells treated with PBS (**c**), RV (**d**), HRP NPs (**e**), HRP–RGD NPs (**f**) stained by Hoechst 33258 after treatment for 24 h. Images were recorded using a digital camera. *Scale bar* = 20 μm. *Red arrows* represent the nuclei pyknotic cells

Figure 6a shows the cytotoxicity of HP–RGD NPs with a concentration range of 0–200 μg/mL by CCK-8 assay, in which PANC-1 cell viability was kept above 90%. It was suggested that levels of RV carrier HP–RGD NPs below 200 μg/mL had no significant cytotoxicity. In addition, the anti-cancer effect of HRP–RGD NPs in vitro was also evaluated. Figure 6b shows that free RV, HRP NPs, and HRP–RGD NPs at RV concentrations ranging between 0 and 50 μg/mL induced a decrease in cell viability through a dose-dependent manner. When compared to free RV and HRP NPs, HRP–RGD NPs can result in greatest decline in cell viability under all test concentrations, likely due to the stability and RGD targeting effects of HRP–RGD NPs to PANC-1 cells [30, 31]. Moreover, the nuclei of PANC-1 cells treated by free RV, HRP NPs, and HRP–RGD NPs (all at 30 μg/mL RV) were stained by Hoechst 33258 and observed under confocal fluorescence microscope. The HRP NPs- and HRP–RGD NPs-treated cells showed the presence of pyknotic nuclei, consistent with RV-treated cells (Fig. 6c–f), indicating that the cell death type is most likely apoptosis [32].

### Blood Circulation and Tumor Biodistribution

**Fig. 7:**
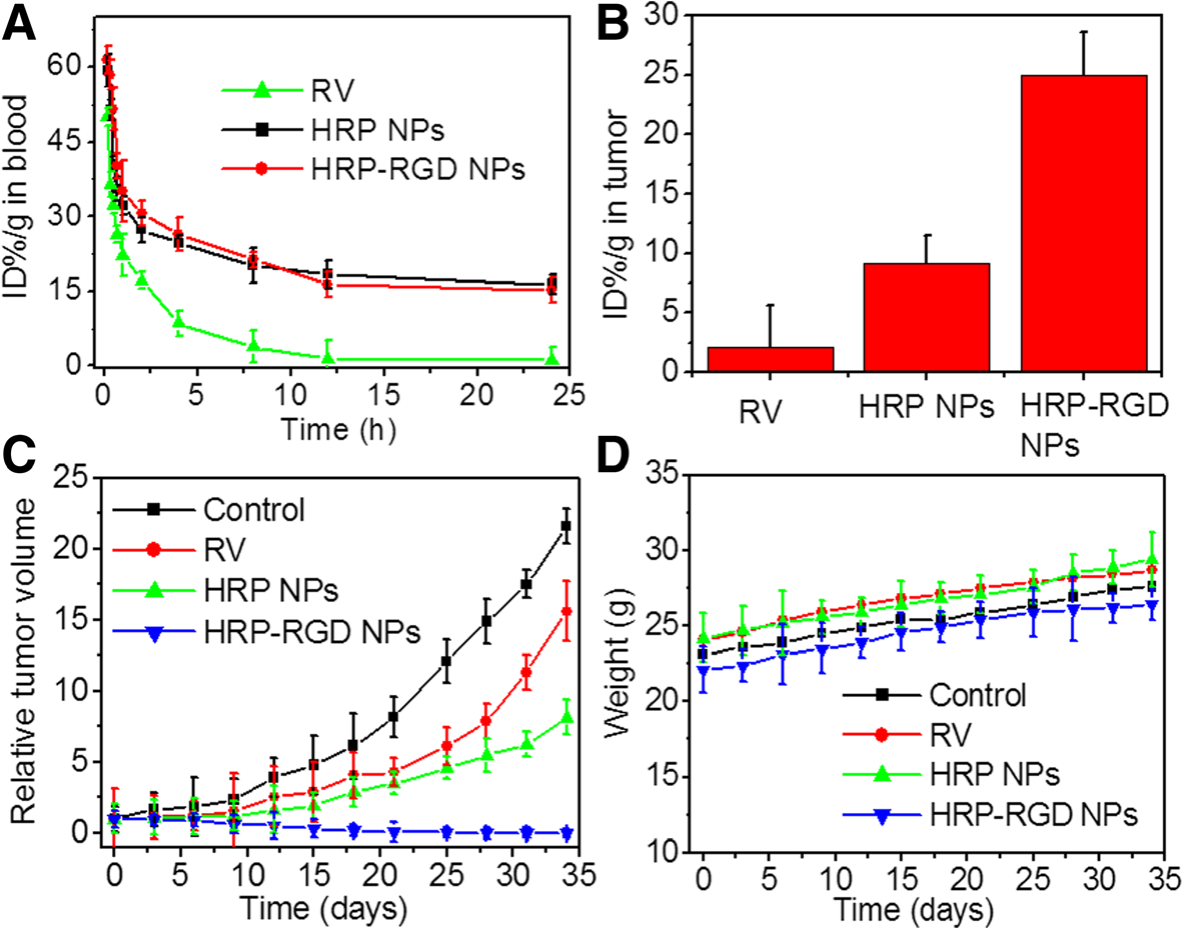
**a** Blood circulation curves of free RV, HRP NPs, and HRP–RGD NPs in mice after intravenous injection determined by the RV absorbance from diluted tissue lysate. **b** Content of RV in tumor at 24 h post-treatment with RV, HRP NPs, and HRP–RGD NPs. **c** The relative tumor volume of tumor-bearing mice after intravenous injection with saline (control), RV, HRP NPs, and HRP–RGD NPs. **d** Body weight of tumor-bearing mice after intravenous injection with saline (control), RV, HRP NPs, and HRP–RGD NPs

Figure 7a shows the blood circulation time of free RV, HRP NPs, and HRP–RGD NPs after intravenous injecting into mice. It can be seen that HRP NPs and HRP–RGD NPs have nearly the same half-life time (*t*_1/2_) of 7.5 ± 0.5 h and *t*_1/2_ = 6.57 ± 0.9 h, respectively. While free RV was quickly removed from the blood circulating system and *t*_1/2_ = 1.21 ± 0.09 h. HRP–RGD NPs prolonged the blood circulation time of RV, approximately increasing 5.43-fold (*t*_1/2_). Furthermore, 24 h post-injection with the nanoparticles, the content of RV in the tumor tissue of the HRP–RGD NPs-treated group was approximately 3.01- and 8.1-fold higher than that of the HRP NPs- and free RV-treated groups, respectively (Fig. 7b). These results indicate that HSA and PEG encapsulation could prolong the circulation time to decrease the elimination of RV and show a significant selective accumulation performance in tumor tissue [33, 34], likely because of the enhanced permeability and retention (EPR) and the RGD targeting effect [35].

### In Vivo Anti-cancer Efficacy

**Fig. 8:**
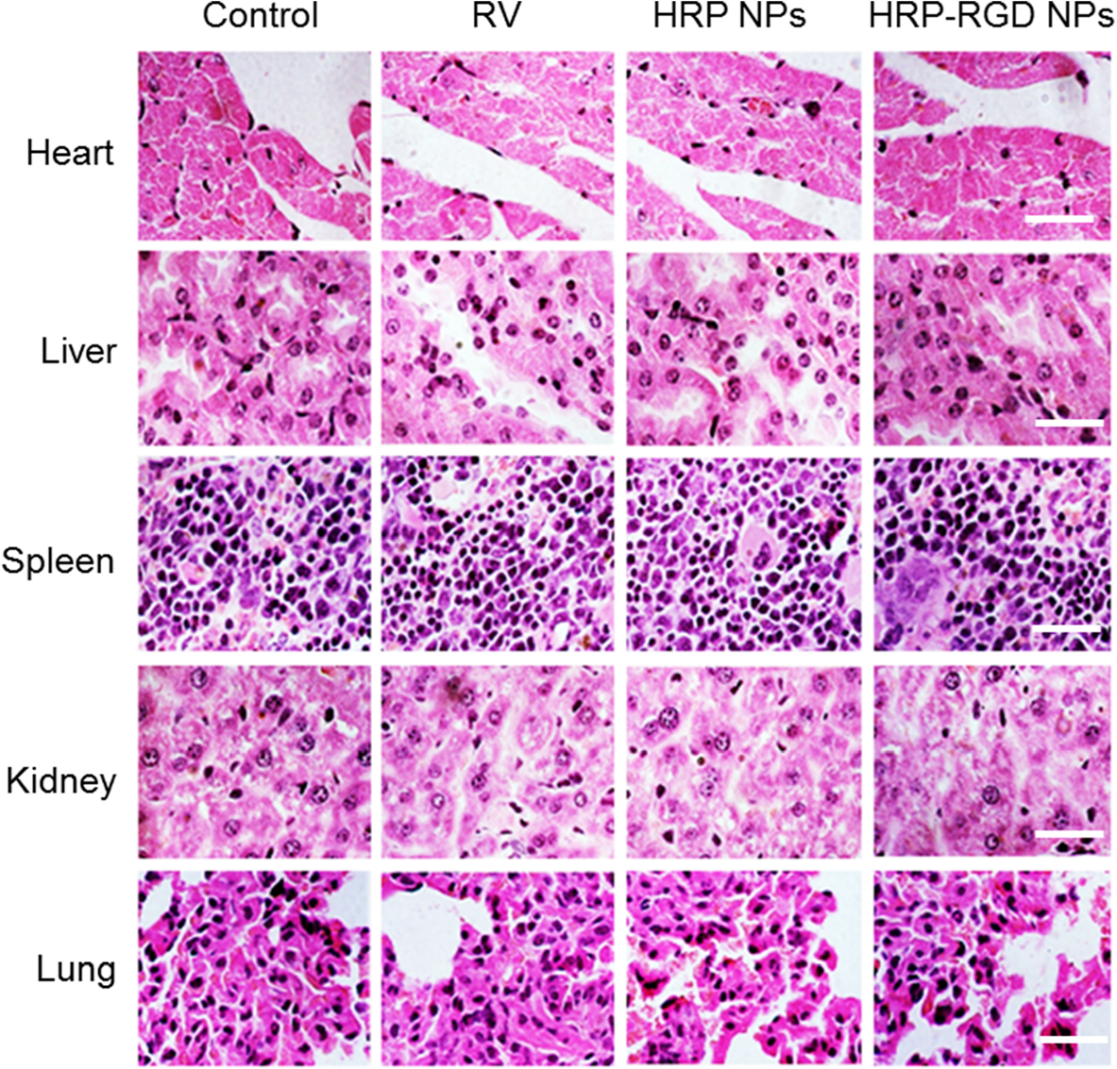
The representative HE staining images of the heart, liver, spleen, kidney, and lung. *Scale bar* = 100 μm

Figure 7c shows the anti-cancer efficiency of free RV, HRP NPs, and HRP–RGD NPs at the same concentration of RV after intravenous tail injection. As a control, mice were treated with saline. HRP–RGD NPs-treated mice showed a significantly suppressed tumor growth and no relapse after 35 days treatment. While free RV, HRP NPs treated groups, similar to the control group, showed ever-increasing tumor growth. As expected, the body weight of the mice in all groups did not decline significantly over 35 days treatment (Fig. 7d). Moreover, the in vivo systemic toxicity was further evaluated by H&E staining of the major organs (heart, liver, spleen, lung, and kidney) after 35 days treatments (Fig. 8). No noticeable tissue toxicity or abnormality was found in the corresponding tissue H&E staining images of all tested groups, which further guaranteed the in vivo safety of HRP–RGD NPs for biomedical applications.

## Conclusions

In summary, we have illustrated how HRP–RGD NPs can be used as a highly effective pancreatic tumor targeting therapeutic agent. It was demonstrated that HRP–RGD NPs exhibited improved colloidal stability and biocompatibility in vitro compared to free RV. RGD as a target molecule promoted the highly efficient cell uptake of HRP–RGD NPs. With the presence of PEG and HSA, HRP–RGD NPs showed a significantly prolonged circulation time that can overcome the short blood circulation of free RV. Based on the RGD targeting, the content of HRP–RGD NPs in tumor tissue was more than that of free RV and HRP NPs. Moreover, in vitro and in vivo studies showed that compared to free RV and HRP NPs, HRP–RGD NPs feature an excellent anti-cancer effect likely induced by apoptosis. At last, HRP–RGD NPs showed high biocompatibility and no significant systemic toxicity in vivo over 35 days treatment. These results demonstrate that HRP–RGD NPs can be promising tumor chemotherapy agent in future biomedical applications.

## Abbreviations

CCK-8: Cell counting kit-8
FBS: Fetal bovine serum
FITC: Fluorescein isothiocyanate
HSA: Human serum albumin
PBS: Phosphate buffer
PEG: Polyethylene glycol
RGD: Arginine–glycine–aspartate
RV: Resveratrol
